# A stochastic automaton shows how enzyme assemblies may contribute to metabolic efficiency

**DOI:** 10.1186/1752-0509-2-27

**Published:** 2008-03-25

**Authors:** Patrick Amar, Guillaume Legent, Michel Thellier, Camille Ripoll, Gilles Bernot, Thomas Nystrom, Milton H Saier, Vic Norris

**Affiliations:** 1 Epigenomics Programme, genopole^®^, 91000 Evry, France; 2 Laboratoire de Recherches en Informatique, Université Paris Sud & CNRS UMR 8623, 15 avenue George Clémenceau, 91405 Orsay, Cedex, France; 3 Laboratoire d'Assemblages moléculaires: modélisation et imagerie SIMS, Faculté des Sciences de l'Université de Rouen, 76821 Mont Saint Aignan Cedex, France; 4 Department of Cell and Molecular Biology, Goteborg University, Medicinaregatan 9C, 413 90 Goteborg, Sweden; 5 Division of Biological Sciences, University of California at San Diego, La Jolla, CA 92093-0116 USA

## Abstract

**Background:**

The advantages of grouping enzymes into metabolons and into higher order structures have long been debated. To quantify these advantages, we have developed a stochastic automaton that allows experiments to be performed in a virtual bacterium with both a membrane and a cytoplasm. We have investigated the general case of transport and metabolism as inspired by the phosphoenolpyruvate:sugar phosphotransferase system (PTS) for glucose importation and by glycolysis.

**Results:**

We show that PTS and glycolytic metabolons can increase production of pyruvate eightfold at low concentrations of phosphoenolpyruvate. A fourfold increase in the numbers of enzyme EI led to a 40% increase in pyruvate production, similar to that observed *in vivo *in the presence of glucose. Although little improvement resulted from the assembly of metabolons into a hyperstructure, such assembly can generate gradients of metabolites and signaling molecules.

**Conclusion:**

*in silico *experiments may be performed successfully using stochastic automata such as HSIM (Hyperstructure Simulator) to help answer fundamental questions in metabolism about the properties of molecular assemblies and to devise strategies to modify such assemblies for biotechnological ends.

## Background

There is evidence from across the phyla that enzymes are often associated with one another into assemblies or metabolons in which metabolites may or may not be channeled directly from one enzyme to the next in the pathway. Investigation of glycolytic metabolons responsible for the metabolism of glucose has a long and controversial history [1,2], but it seems clear that in eukaryotes several of the glycolytic enzymes can form oligomers [3] and associate with other glycolytic enzymes [4,5]. In the model prokaryote, *Escherichia coli*, the glycolytic pathway has been isolated as a large equimolar multi-enzyme complex in which metabolites were sequestered [6,7]. There is also evidence that enzymes may be assembled into much larger *hyperstructures *[8], as in the case of cellulolytic enzymes in bacteria such as *Acetivibrio cellulolyticus *[9] or compartmentalized into organelles such as glycosomes as in the case of glycolytic enzymes in trypanosomes [10], or associated with the tubulin cytoskeleton, as in the case of many glycolytic enzymes in mammalian cells [11]. The actual advantages – or lack of them – conferred by metabolons and hyperstructures on metabolic efficiency are unclear. What happens to efficiency when enzymes responsible for import are restricted to a patch of membrane, or when cytoplasmic enzymes are bound to membrane transporters, or when metabolons are brought together in a hyperstructure? If quantitative answers to these questions could be obtained rapidly and cheaply *in silico*, it would be of value not only to the understanding of existing systems but also to the construction of engineered systems. The development of programs that allow genetic engineers to know in advance which constructions are worth making is therefore biotechnology's equivalent of the quest for the Holy Grail.

Simulation with stochastic automata and multi-agent systems is an attractive alternative to differential equations for studying the diffusion and interaction of the many different enzymes and metabolites in cells which may be present in either small or large numbers [12-18]. A stochastic automaton, HSIM (for Hyperstructure Simulator), has been developed and an early version used to model the assembly of cytoskeletal filaments in a virtual cell [19]. In extending HSIM to the analysis of the assembly, movement and disassembly of large numbers of molecules and macromolecules, we have chosen to base our studies very loosely on two pathways that continue to be intensely studied in *E. coli*: the phosphoenolpyruvate:sugar phosphotransferase system (PTS), which is responsible for sensing and importing sugars such as glucose and which supplies sugar phosphates to a second intensely studied pathway, the glycolytic pathway [20,21]. Here, we use HSIM to determine quantitatively the effects of metabolon and hyperstructure formation on a modified version of these systems in which glycolysis is truncated.

## Results

### Equal numbers of enzymes in varying conditions of association

**Figure 1:**
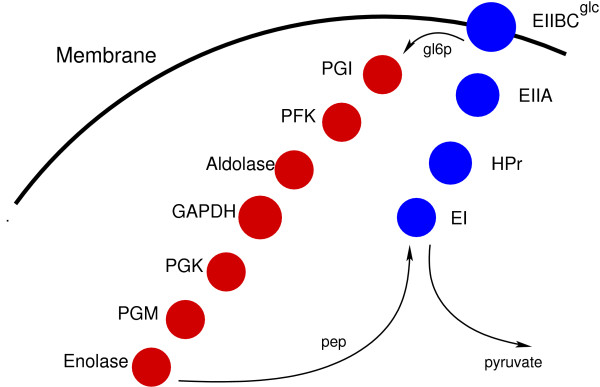
**Schematic version of a modified PTS and glycolysis**. The relationships between the enzymes in a modified version of the PTS and glycolysis in *E. coli *are shown. EIIBC (IICB^Glc^), *ptsG*; EIIA (IIA^Glc^), *crr*; HPr, *ptsH*; EI, *ptsI*; phosphoglucose isomerase, PGI; phosphofructokinase, PFK; aldolase, *fbaA*; glyceraldehyde 3-dehydrogenase A complex, GAPDH; phosphoglycerate kinase, PGK; phosphoglycerate mutaseA, PGM.

**Figure 2:**
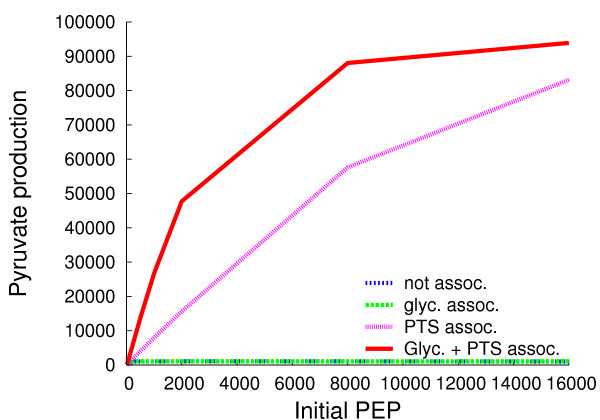
**Pyruvate production**. The numbers of pyruvate molecules produced are plotted against initial PEP concentrations after 40 s in the simulation when the enzymes are either associated in hyperstructures or free. A broad range of initial PEP concentrations is shown. Note that pyruvate kinase, a glycolytic enzyme that converts PEP into pyruvate, is absent from our system, and this conversion is only performed by EI.

**Figure 3:**
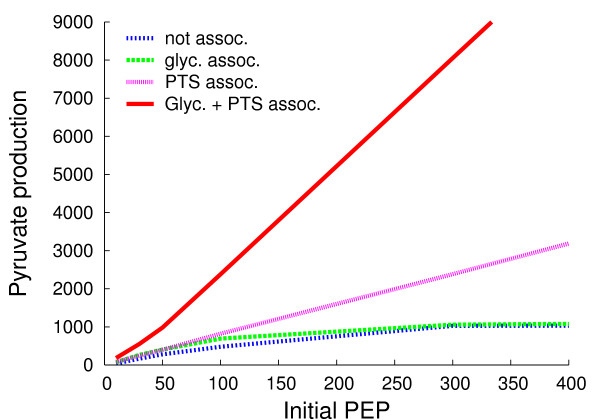
**Pyruvate production**. The numbers of pyruvate molecules produced are plotted against initial PEP concentrations as in Fig. 2 and are shown for low initial PEP concentrations.

The extent to which the assembly of enzymes into metabolons confers greater efficiency in a modified version of glycolysis and the PTS (see Figure 1 for the enzymes simulated) was the first question we addressed after testing HSIM (see Methods). Glycolysis is modified because the real glycolytic pathway yields two PEPs per G6P which are converted into pyruvate by pyruvate kinase but it is not known how much of this PEP is available for the PTS *in vivo*, let alone how much is available in an individual cell as opposed to a population of cells. An initial presence of PEP is required to prime the system. In conditions in which only one PEP per glucose imported is available for the PTS, the maximum rate of functioning is determined by the initial concentration of PEP (Figure 2, 3 and Additional Table 1). This is because each PEP is transformed into a pyruvate and donates a phosphate to allow generation of another PEP. The numbers of pyruvate molecules produced are given after 40 s simulation when the number of pyruvate molecules is a linear function of time (it should be noted that the stationary state of production of pyruvate is attained after two to three seconds in all cases shown here). Consider first conditions in which the number of PEP molecules is 2000 and in which metabolons allow the products of one enzyme to be released in the vicinity of the adjacent one; that is, channeling is not obligatory, and metabolites can escape from the metabolon. When the glycolytic enzymes are assembled into spatially separate glycolytic metabolons, the quantity of pyruvate made is no greater than when these enzymes are separate, but when the PTS enzymes are assembled into PTS metabolons, the quantity of pyruvate made is fifteen times greater (Figure 2). In the latter conditions, glycolysis then becomes limiting as shown by the fact that when all the enzymes are in separate metabolons, the quantity of pyruvate made reaches its maximum and is forty-four times greater. This pattern is conserved for all initial concentrations of PEP over 300. In general, pyruvate production is highest at the highest initial concentrations of PEP. When the PTS enzymes are free, and when the initial concentrations of PEP are high (≥ 2000), the yield of pyruvate is much less than that of the PEP. This is because the flow of phosphate through the PTS is too slow, and the enzyme EI is constantly phosphorylated so that it cannot fully exploit the available pool of PEP. At low initial numbers of PEP molecules (< 300), both glycolytic and PTS metabolons are advantageous (Figure 3 and Additional Table 1). This is because both sets of enzymes are under-employed, and improving the efficiency of either glycolysis or the PTS allows the enzymes in the other set to function. It might be argued that the concentrations of PTS and glycolytic enzymes are higher in real cells by an order of magnitude and that this is a very important factor in the efficiency. However, decreasing the volume of the cell 8-fold while retaining the same numbers of enzymes so as to make concentrations similar to those of the real PTS did not result in qualitative changes in the results (see Table 1). That said, the levels of pyruvate production for the free enzymes are an order of magnitude higher at all initial PEP values when these enzymes are concentrated in the smaller volume.

**Table 1:** Pyruvate production at high concentration. The numbers of pyruvate molecules produced are given after 40 s in the simulation when the enzymes are either associated in hyperstructures or free as in Additional Table 1 but at an 8-fold higher concentration (the volume was reduced by this factor).

**Initial PEP**	**10**	**30**	**100**	**300**	**500**	**1000**	**2000**
Not assoc.	573	1662	5287	10989	11068	10755	10082
Gly. assoc.	909	2770	7571	11427	11296	10832	10413
PTS assoc.	748	2343	7838	22316	35503	61753	95946
Gly + PTS	1359	5357	18326	49629	68867	97755	113739

**Figure 4:**
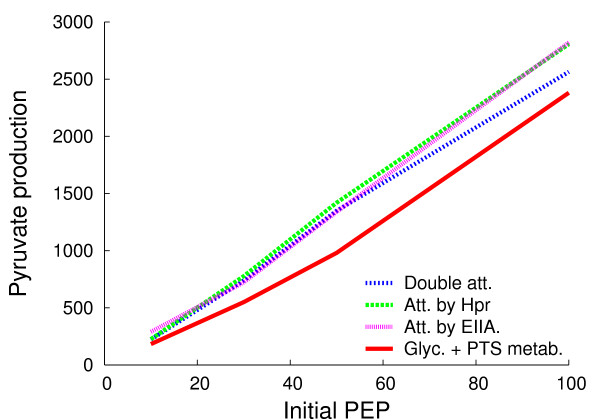
**Pyruvate production with metabolon colocalization**. The complementary metabolons in Fig. 2 were attached at different positions to make pairs. Att., attachment of the first enzyme in glycolysis, PGI to a PTS enzyme either EIIA or HPr; Double Att. attachment of PGI to EIIA plus attachment of enolase to EI; Sep., separate glycolytic and PTS metabolons.

Since confining enzymes to complementary metabolons increases efficiency, we asked whether the colocalization of complementary metabolons would increase efficiency still further. To explore the effects of the colocalization of glycolytic and PTS metabolons as individual pairs, we attached the first enzyme in our version of the glycolytic pathway, PGI, to EIIA and, in a separate experiment, to HPr (we did not study PGI attachment to EIIBC because PGI then sterically hinders the import of glucose in the program; attachment further down the PTS largely avoids this artifact). The attachment of the glycolytic metabolon to the PTS metabolon at EIIA or HPr improved the yield of pyruvate at the lower initial concentrations of PEP by up to 150% (compared with separate glycolytic and PTS metabolons) but had no significant effect over 1000 molecules of initial PEP which is when the PTS can import sufficient glucose for colocalization of the two metabolons to be no longer of an advantage (Figure 4 and Additional Table 1). In the above colocalization studies, enolase, the final enzyme in our truncated glycolytic metabolon, could release PEP far from EI the first enzyme in the PTS. We therefore tested the effect of attaching enolase to EI in conditions in which PGI was also attached to EIIA (for technical reasons, only 58 metabolons were studied). This double attachment gave a slightly lower yield of pyruvate compared with the single attachment cases. Hence colocalization of complementary metabolons can lead to an increase in efficiency in some conditions but this is modest in comparison with the increase conferred by metabolon formation itself.

### Altering numbers of enzymes in varying conditions of association

It is conceivable that the encounters between the substrate and the enzyme on the end of the metabolon are limiting. To test this (Table 2), we increased the number of EI fourfold since this is the increase observed when the synthesis of this enzyme is induced in the presence of glucose (note that where PTS metabolons were formed, the extra EI diffused freely). Increased dosage of EI had no effect in the presence of PTS metabolons since diffusion of PEP to the metabolon is essentially limited by the relatively rapid rate of diffusion of PEP itself. Increased dosage did, however, have an effect when the PTS enzymes were limiting and, as shown in the column for 500 initial molecules of PEP, resulted in a 40% increase in the yield of pyruvate. At the lower concentration of 50 initial PEP molecules, there was no significant advantage to be gained from the increase in EI in any condition whilst at the higher concentration of 1000 initial PEP molecules, most of the new EIs are phosphorylated and again increased the yield of pyruvate 40% when the PTS enzymes were limiting. The fact that the increased EI dosage makes a difference only when the PEP concentration is high or when the PTS enzymes diffuse freely (so PTS enzymes become limiting) makes sense, since in a complex, or when PEP is diffusion limiting, there should be little benefit from having extra EI outside the metabolon. Thus, the results conform to expectation if the PTS is truly rate limiting for overall glucose metabolism. These results also help answer the question of how many rate-limiting steps there are. The fact that the overall rate increases when the glycolytic enzymes are in a metabolon indicates that there can be more than one rate-limiting step. This might be related to phosphofructokinase being an important allosterically controlled enzyme with a key role in the flux of carbon through glycolysis. However, increasing the level of phosphofructokinase fourfold had no significant effect.

**Table 2:** Pyruvate production with increasing numbers of EI. Comparison between the production of pyruvate in different metabolon conditions when the numbers of EI are increased fourfold. (other details essentially as in Figure 2).

**Initial PEP:**	**50**	**50**	**50**	**500**	**500**	**500**	**1000**	**1000**	**1000**
	EI × 4 PFK × 4			EI × 4 PFK × 4			EI × 4 PFK × 4		

No assoc.	279	294	284	1036	1439	1163	1045	1432	1212
Gly. assoc.	405	455	417	1096	1446	1058	1054	1443	1104
PTS assoc.	401	394	422	3990	3995	4273	7982	7937	8732
Gly + PTS	981	1001	1046	13699	14025	13723	26752	26230	26088
Attach EIIA	1340	1364	1402	14129	13964	14224	26629	26634	26673

### Effects of restricting enzymes to a region of the membrane/cytoplasm

Bacteria are now known to be highly structured and it is interesting to investigate the efficacy conferred by the assembly of metabolons into a hyperstructure. We therefore adapted the program so that only glycolytic enzymes function with the exception of Enzyme IIBC which imports glucose and converts it into P-Glu spontaneously (i.e., without the need for the rest of the PTS). In all cases, the Enzyme IIBC species were restricted to a small patch of membrane while the glycolytic metabolons were positioned 4 nm away. As explained above, direct attachment to EIIBC was avoided because this would have hindered the import of glucose in the present version of the program. It should be noted that PEP accumulates in this experiment because it is consumed by neither EI nor pyruvate kinase.

There is no significant gain in efficacy when the enzymes are in a hyperstructure in which there is no channeling versus the case when the enzymes are free (Table 3). Indeed, at the three rates of glucose import tested, the PEP production in a hyperstructure comprising 60 metabolons without channeling is similar to that of the same number of freely diffusible enzymes. Hence, at least under these conditions, the prediction that a metabolite that escapes from a metabolon could be captured by a neighboring metabolon is false. Not surprisingly, channeling between the glycolytic enzymes in the metabolons gives the highest yield of PEP. It should be noted that at the highest rate of import, more glucose is entering than glycolysis can consume, and a stationary state is not attained. The concentration of P-Glu is another interesting parameter that could be studied.

**Table 3:** Glycolytic metabolons in hyperstructures. PEP production after 10 s when the EIIBC enzymes are in a patch and i/when the glycolytic enzymes diffuse freely or ii/when these enzymes are assembled into 60 glycolytic metabolons in a hyperstructure with or without channeling (a single enzyme space was left between the patch of EIIBC enzymes that import and phosphorylate glucose and the glycolytic hyperstructure).

**Glucose per sec.**	**0.1**	**1**	**10**
Free enzymes	16	216	2131
Hyperstructure no channeling	25	253	2178
Hyperstructure + channeling	55	428	4097

**Figure 5:**
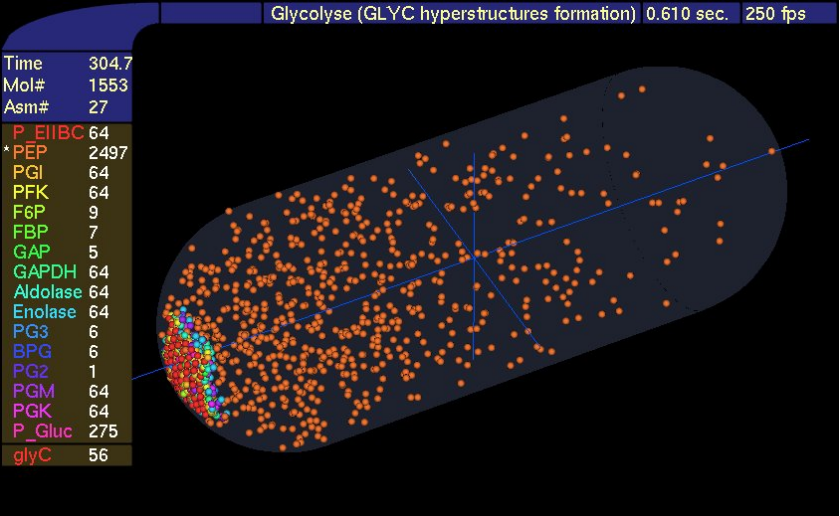
**Gradient of PEP**. The glycolytic hyperstructure containing 56 metabolons was localized to a pole, and the distribution of PEP and the enzymes were displayed. A freely diffusing enzyme consumed PEP. GlyC is the number of glycolytic metabolons, other abbreviations as in Fig. 1.

The association of enzymes into metabolons is a potentially powerful way of generating signals [22] and there may be additional advantages in confining metabolons to a few hyperstructures, for example, in generating gradients of metabolites or signaling molecules. To illustrate this, we positioned the patch of EIIBCs and the glycolytic hyperstructure described above at a cell pole and allowed each molecule to be consumed in a reaction catalyzed by a freely diffusible enzyme. Under these conditions, there is a broad range of values of the coefficients of production, diffusion and consumption that generate an easily observable gradient (Figure 5). Where the receptors of signaling molecules are themselves confined to a region, such gradients may lead to the concentration of the molecule exceeding a threshold and hence be of physiological importance; HSIM offers the possibility of investigating these situations.

## Discussion

There is increasing evidence that in both eukaryotic and prokaryotic cells, enzymes are organized into higher order assemblies. In metabolism, such assemblies may take the form of metabolons [23] which may themselves be associated into still higher order hyperstructures [24,25]. In signaling, chemotaxis proteins may be organized into arrays in the poles of certain bacteria [26], while in eukaryotes, signal transduction proteins may be assembled into transducons or signalosomes [27]. Exploring and exploiting the consequences of such organization is a major challenge. What are the advantages, if any, of the colocalization of successive enzymes in a pathway? Can these advantages be quantified? What are the effects of increasing the number of copies of a particular enzyme or of the efficiency of the reaction that it catalyzes? What happens when enzymes responsible for import are restricted to a patch of membrane or when cytoplasmic enzymes are bound to membrane enzymes or transporters? To help provide quantitative answers to such questions, we have developed a stochastic automaton, HSIM [19], to simulate realistically the diffusion of metabolites and enzymes in both the membrane and the cytoplasm, the reactions between enzymes and their substrates, and interactions between enzymes leading to their assembly into metabolons and hyperstructures. For a typical workstation, HSIM takes 3 minutes to simulate 1 minute of real time for a cell, irrespective of its size, containing a total of 1000 molecules (or enzymes), irrespective of the number of classes of these molecules.

The system that we have investigated with HSIM is a simplified one loosely based on the PTS and glycolysis in *E. coli *which, although intensely studied for many years, is still imperfectly understood. For example, how many rate-limiting steps are there, and how might these be related to the formation of metabolons? Our version of glycolysis has 7 enzymes that act successively to produce only one PEP from each G6P. As in the real PTS, our version has 4 enzymes that pass a phosphate group from one another to the final membrane-bound enzyme, EIICB^Glc^, that imports and phosphorylates glucose to make G6P and completes the loop in the synthesis of one PEP from one G6P. This system has the advantage of avoiding the complication of the rate of PEP production being non-linear due to both synthesis of two PEP molecules per G6P and consumption of PEP also depending on pyruvate kinase (which metabolizes it into pyruvate). Enolase, the final glycolytic enzyme in our version, catalyzes the production of pyruvate from PEP and transfers a phosphate to EI, the first enzyme in the PTS. Pyruvate production is used as a measure of the efficacy of the system. Enzymes can either diffuse freely and release their products immediately or be in metabolons. Enzymes in metabolons can either release their products immediately to diffuse away or channel them obligatorily to the next enzyme in the pathway, provided this recipient enzyme is itself in a state to receive the metabolite (i.e., has passed its own product on).

The initial concentrations of PEP at which increased efficiency is conferred by assembly of either the PTS or the glycolytic enzymes into separate metabolons are readily revealed by HSIM. Moreover, descriptions that would otherwise be qualitative can be turned into quantitative ones. For example, at intermediate initial levels of PEP (around 500 molecules), the PTS is limiting while glycolysis is not, and the formation of PTS metabolons increases pyruvate production fourfold. At low levels of PEP (< 300), both glycolytic and PTS metabolons are advantageous, and the formation of both sets of metabolons can increase pyruvate production up to eightfold. We then tested the prediction that attaching the PTS and glycolytic metabolons to form pairs would increase efficiency relative to these metabolons that are not physically associated. A relatively modest increase of 150% was observed for some PEP concentrations below 1000 per cell, and no increase was observed over that. HSIM can be used to investigate the possibility that a particular enzyme is rate-limiting in a pathway, and that increasing the number of copies of the enzyme will improve efficiency (but see [28]). To illustrate this, we increased the number of EI fourfold, which corresponds to the increase observed when glucose is added. This resulted in a 40% increase in pyruvate formation under conditions where the PTS enzymes were already limiting. The association of enzymes into hyperstructures much larger than metabolons either exists already or can be created by genetic engineering by modifying enzymes so that they associate either with themselves into homopolymers or with other enzymes into heteropolymers or with cytoskeletal structures or with membrane domains. Surprisingly, there was little advantage to be gained in terms of efficiency of pyruvate production from a hyperstructure unless channeling was allowed. This doubled efficiency. It is, of course, conceivable that the advantage of grouping enzymes into hyperstructures lies elsewhere in, for example, signaling [29]. We therefore positioned a hyperstructure at a pole and explored the consequences on the distribution of PEP under conditions in which PEP is consumed in a reaction catalyzed by a freely diffusible enzyme. This showed that a gradient of PEP can be generated by a broad range of values of different parameters; in other words, if enzymes are altered so as to assemble into a hyperstructure, it is easy to form a gradient.

## Conclusion

In sum, our results show how HSIM may help answer fundamental problems and evaluate biotechnological objectives. Here we have applied HSIM to the analysis of a simplified model system in steady state conditions. However, it may also prove useful where parameters such as numbers of enzymes and substrates fluctuate. Continued development of HSIM and other stochastic automata and multi-agent systems should show that the dream of meaningful experiments *in silico *is not a pipedream.

## Methods

### The stochastic automaton

**Figure 6:**
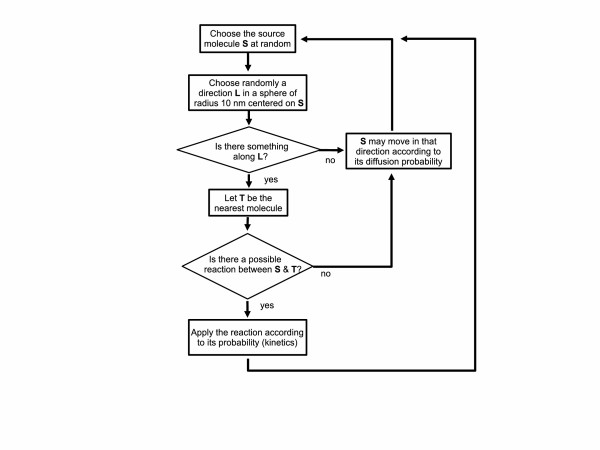
Overview of HSIM.

The simulator, HSIM, is a stochastic automaton driven by reaction rules between molecules (for an overview see Figure 6; a compiled version is available for Windows at [30] and for Linux at [31]). In essence, each molecule is represented by a record that includes its type, its position, and a list of links to certain other molecules. HSIM keeps track of each molecule in real time from the computer point of view. The basic principle is that time is sliced into consecutive steps or *generations*, and in each generation the rules are applied to every molecule. These rules mimic the chemical reactions between molecules in a real system. The generation time is set to 100 microseconds, which corresponds to the average time for a protein to move a distance of 10 nanometers (of the order of its diameter) *in vivo *[32]. Metabolites diffuse ten times faster than proteins in HSIM to take account of their smaller size, however, for convenience, they are represented in HSIM by a sphere of the same size. During a generation, the following processes are applied to all the molecules: the source molecule S is chosen at random (in order to avoid systematic artifacts); the presence of a target molecule, T, is checked for in close proximity to S by searching in a sphere of radius 10 nm centered on S along a random direction (two angles in the 3D space); if another molecule intersects this line and if a reaction rule exists between a molecule of type S and a molecule of type T, this rule is applied, according to a probability representing the reaction kinetics (if not, molecule S may move to the empty location L, according to a probability representing the diffusion speed). When all the molecules in the cell have been processed, the generation is completed and a new one begins. One important point is that in HSIM the computer time is proportional to the total number of molecules and not to the size of the simulated space or the number of types of molecules. There are four kinds of interaction rules in HSIM between two molecules: **Reaction**: S reacts with T to produce two other types of molecules S' and T'; **Association**: S binds to T to produce the complex S-T; **Dissociation**: the complex S-T dissociates into individual molecules S and T; **Catalysis**: the complex S-T is transformed into S'-T'.

At the start of the simulation, the cytoplasmic enzymes are grouped together in blocks and the integral membrane enzymes in a patch. To form spatially distant metabolons, the enzymes are allowed to diffuse randomly before they are given an affinity for one another, whereas to form a single hyperstructure, they are given affinities before being allowed to diffuse. A hyperstructure itself can move with a speed that is inversely related to its size.

### Testing HSIM

**Figure 7:**
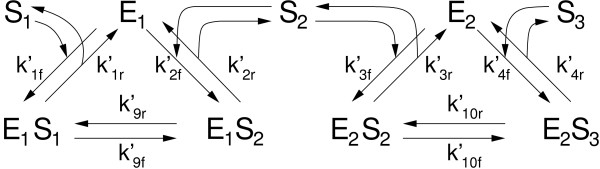
**The two-enzyme test model**. The first enzyme, E_1_, binds the initial substrate, S_1_, to form the enzyme-substrate complex, E_1_S_1_. Within this complex, E_1 _transforms S_1 _into its product, S_2_, resulting in the transformation of E_1_S_1 _into E_1_S_2_, and then E_1_S_2 _liberates S_2_, thus regenerating E_1_. In a similar manner, the second enzyme E_2 _binds S_2_; transforms S_2 _into S_3 _and finally releases the final product, S_3_. The k'_if _and k'_ir _are the forward and reverse rate constants; their numbering, i, has been chosen in such a way as to be consistent with that of previous publications.

To test HSIM, we compared the results generated by HSIM with those generated by a system of ordinary differential equations in the case of two sequential enzymes of the Michaelis-Menten type catalysing the transformation of an initial substrate S_1 _into a final product S_3 _(Figure 7) [33]. This model comprises two reaction circuits, where the first and second circuits correspond to the activity of the first and second enzymes, E_1 _and E_2_, respectively. Six ordinary differential equations were obtained by writing down the mass balance of the six independent species involved, i.e.

dE_1_/dt = k'_1r_·E_1_S_1 _+ k'_2r_·E_1_S_2 _- (k'_1f_·S_1 _+ k'_2f_·S_2_).E_1_

dE_2_/dt = k'_3r_·E_2_S_2 _+ k'_4r_·E_2_S_3 _- (k'_3f_·S_2 _+ k'_4f_·S_3_).E_2_

dS_1_/dt = k'_1r_·E_1_S_1 _- k'_1f_·E_1_.S_1 _

dS_2_/dt = k'_2r_·E_1_S_2 _+ k'_3r_·E_2_S_2 _- (k'_2f_·E_1 _+ k'_3f_·E_2_).S_2_

dS_3_/dt = k'_4r_·E_2_S_3 _- k'_4f_·E_2_.S_3_

dE_2_S_3_/dt = k'_4f_·E_2_.S_3 _+ k'_10f_·E_2_S_2 _- k'_4r_·E_2_S_3 _- k'_10r_·E_2_S_3_

which together with the 3 mass balance algebraic equations:

E_1 _+ E_1_S_1 _= E_1t_

E_2 _+ E_2_S_2 _= E_2t_

S_1 _+S_2 _+ S_3 _+ E_1_S_1_+ E_1_S_2_+ E_2_S_2_+ E_2_S_3_= S_1 _^0 ^+ S_2 _^0 ^+ S_3 _^0^

allow calculation of the concentrations of the 9 species involved in the system: E_1_, E_2_, S_1_, S_2_, S_3_, E_1_S_1_, E_1_S_2_, E_2_S_2 _and E_2_S_3 _using the initial conditions S_1 _(0) = S_1 _^0^, S_2 _(0) = S_2 _^0^, S_3_(0) = S_3 _^0^, E_1 _(0) = E_1t_, E_2 _(0) = E_2t_, the initial concentrations of the other species being zero. In these equations, k'_if_, k'_ir_, are the forward and reverse rate constants, E_1t _and E_2t _are the constant total concentrations of the enzymes, S_1 _^0^, S_2 _^0 ^and S_3 _^0 ^are the initial concentrations of S1, S2 and S3 and t is the time. Moreover, the rate constants have to satisfy the principle of detailed balance, i.e.

(k'_1f_·k'_2r_·k'_3f_·k'_4r_·k'_9f_·k'_10f_)/(k'_1r_·k'_2f_·k'_3r_·k'_4f_·k'_9r_·k'_10r_) = K

in which K is the equilibrium constant of the overall reaction of S_1 _into S_3_.

Numerical resolutions were performed using the MAPLE 9.5 software to solve the above non-linear ODE system with the given initial conditions.

**Figure 8:**
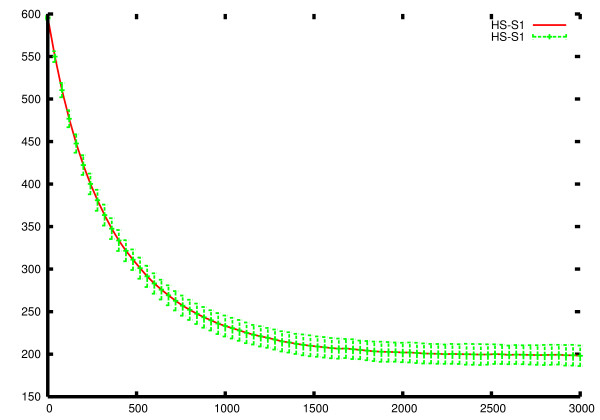
**Average curve and standard deviation**. This curve shows the mean values (red curve) and the standard deviations (green bars) of the concentration of S_1 _calculated at each point from 600 different runs of HSIM.

HSIM is a stochastic automaton system hence many independent HSIM simulations for a single given set of initial conditions were performed to obtain statistically significant values. At each time point, we calculated the mean value of S_1 _concentration as well as the standard deviation (Figure 8). We also calculated, at each time point, the relative difference between the mean values of S_1 _concentration obtained after 400 and 600 runs, which were less than 10^-4^, and those obtained after 500 and 600 runs, which were less than 10^-5^. We therefore estimate that 600 runs are sufficient to approximate the mean values of S_1 _concentration which would be obtained after an infinite number of runs. These mean values should be identical to those given by the ODE system. In order to set up the ODE system, it was necessary to give values to the rate constants corresponding to the parameter values of the HSIM simulations. From the result of the 600 runs, we obtained the equilibrium values of the number of molecules of all the species. These equilibrium values were converted to molar concentrations taking into account the volume of the virtual bacterium. These concentrations were then used to calculate the equilibrium constants of the reactions shown in Figure 7. These are written

K_i _= k'_if_/k'_ir _with i = 1, 2, 3, 4 and 10

We then arbitrarily chose

k'_ir _= 1.τ

and calculated

k'_if _= K_i_.k'_ir_

τ is a time scaling factor calculated to obtain the best fit between the ODE and the HSIM values at each time. This value was obtained by minimization of the sum:

$$\sum_{j=0}^{600}{\left[S_{1,j}(ODE)-S_{1,j}(HSIM)\right]}^{2}$$

**Figure 9:**
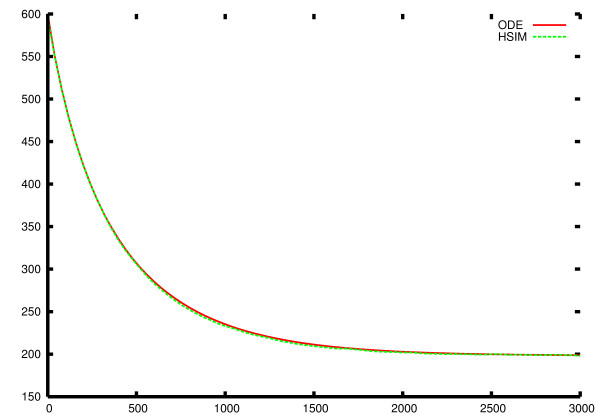
**Comparison of HSIM and ODEs with same initial conditions**. The red continuous line is the solution of the ODE system, while the green dotted line is plotted from the mean values of 600 independent HSIM simulations with the same initial conditions. To compare the two models, the rate constants were calculated as explained in the text.

Where *S*_1,*j*_(*ODE*) and *S*_1,*j*_(*HSIM*) are the S_1 _concentrations given by the ODE system and the HSIM automaton (mean values) at time j. This τ was used *without modification *to test the fit between the ODE and HSIM values for the two other metabolites S2 and S3. This shows that the average values obtained by HSIM correspond to those obtained using ODEs (Figure 9).

Finally, to compare HSIM with a published cellular automaton model, we used the same parameters and values as in a 2-D cellular automaton [15]. HSIM gave exactly the same results as in their Figure 2 (data not shown).

### The simplified metabolic model

Our version of glycolysis acts on glucose-6-phosphate to convert it into phosphenolpyruvate via a pathway containing 7 enzymes (Figure 1). The real glycolytic pathway yields two PEPs per G6P which are converted into pyruvate by pyruvate kinase. To limit our study, pyruvate kinase and triose phosphate isomerase are omitted and, unlike real glycolysis, aldolase splits fructose-6-bisphosphate into one glyceraldehyde-3-phosphate without an accompanying dihydroxyacetone phosphate so that our pathway yields one and not two PEP per G6P. Enolase, in our version of glycolysis, is the final enzyme and catalyzes the production of PEP. Interactions between successive enzymes in the PTS result in a reaction catalyzed by EI that entails the phosphate from PEP being transferred to HPr to EIIA to EIICB^Glc^. Membrane-bound EIICB^Glc ^then imports and phosphorylates glucose to yield G6P as the substrate for glycolysis. The initial PEP concentration is an important parameter here because PEP is the basis for the phosphate transfer that leads to glucose being imported. The spatial position and number of any enzymes and any metabolites, including all metabolic intermediates, can be displayed. The number of PTS and glycolytic enzymes in a real *E. coli *range roughly from about 2000 for IICB^Glc ^to about 40000 for HPr (depending on growth conditions) [12]. Because our objective here is to test general hypotheses rather than those restricted to a specific system such as the PTS where important information is still missing, the initial system explored here has 64 copies of each class of enzyme, irrespective of whether they are membrane-bound or cytoplasmic, and the volume of our model bacterium is a quarter of an *E. coli *growing relatively slowly. The mean volume of this cell is about 1 cubic micron in glucose minimal medium. [Note that simulations were also performed with the same numbers of enzymes but in a volume that was reduced eightfold]. For simplicity, the operation of the system under different conditions is analyzed here only in terms of the numbers of pyruvate molecules formed although details of all other molecules are available.

## Authors' contributions

PA designed HSIM, performed research, analyzed data and wrote the paper. GL used the ODEs and analyzed data. MT designed the ODE research for FDSs and analyzed data. CR initiated multi-agent studies, designed research and analyzed data. GB designed research. TN performed enzyme research. MHS designed research based on the PTS/glycolysis, analyzed data and wrote the paper. VN conceived and coordinated the study and wrote the paper. All authors read and approved the final manuscript.

## Supplementary Material

Additional file 1 **Pyruvate production**. The numbers of pyruvate molecules produced are given after 40 s in the simulation when the enzymes are either associated in hyperstructures or free. Note that pyruvate kinase, a glycolytic enzyme that converts PEP into pyruvate, is absent from our system, and this conversion is only performed by EI. Att., attachment of the first enzyme in glycolysis, PGI to a PTS enzyme. Click here for file
